# Effect of Benfotiamine on Advanced Glycation Endproducts and Markers of Endothelial Dysfunction and Inflammation in Diabetic Nephropathy

**DOI:** 10.1371/journal.pone.0040427

**Published:** 2012-07-06

**Authors:** Alaa Alkhalaf, Nanne Kleefstra, Klaas H. Groenier, Henk J. G. Bilo, Reinold O. B. Gans, Peter Heeringa, Jean L. Scheijen, Casper G. Schalkwijk, Gerjan J. Navis, Stephan J. L. Bakker

**Affiliations:** 1 Department of Internal Medicine, University Medical Center Groningen, University of Groningen, Groningen, The Netherlands; 2 Diabetes Centre, Isala Clinics, Zwolle, The Netherlands; 3 Department of Internal Medicine, Isala Clinics, Zwolle, The Netherlands; 4 Langerhans Medical Research Group, Zwolle, The Netherlands; 5 Department of General Practice, University Medical Center Groningen, University of Groningen, Groningen, The Netherlands; 6 Department of Pathology and Medical Biology, University Medical Center Groningen, University of Groningen, Groningen, The Netherlands; 7 Department of Internal Medicine, University Hospital Maastricht, Maastricht, The Netherlands; Mario Negri Institute for Pharmacological Research and Azienda Ospedaliera Ospedali Riuniti di Bergamo, Italy

## Abstract

**Background:**

Formation of advanced glycation endproducts (AGEs), endothelial dysfunction, and low-grade inflammation are intermediate pathways of hyperglycemia-induced vascular complications. We investigated the effect of benfotiamine on markers of these pathways in patients with type 2 diabetes and nephropathy.

**Methods:**

Patients with type 2 diabetes and urinary albumin excretion in the high-normal and microalbuminuric range (15–300 mg/24h) were randomized to receive benfotiamine (*n* = 39) or placebo (*n* = 43). Plasma and urinary AGEs (*N* ^ε^-(carboxymethyl) lysine [CML], *N* ^ε^-(Carboxyethyl) lysine [CEL], and 5-hydro-5-methylimidazolone [MG-H1]) and plasma markers of endothelial dysfunction (soluble vascular cell adhesion molecule-1 [sVCAM-1], soluble intercellular adhesion molecule-1 [sICAM-1], soluble E-selectin) and low-grade inflammation (high-sensitivity C-reactive protein [hs-CRP], serum amyloid-A [SAA], myeloperoxidase [MPO]) were measured at baseline and after 6 and 12 weeks.

**Results:**

Compared to placebo, benfotiamine did not result in significant reductions in plasma or urinary AGEs or plasma markers of endothelial dysfunction and low-grade inflammation.

**Conclusions:**

Benfotiamine for 12 weeks did not significantly affect intermediate pathways of hyperglycemia-induced vascular complications.

**Trial Regristration:**

ClinicalTrials.gov NCT00565318

## Introduction

Diabetic nephropathy (DN) is a serious complication of diabetes and a leading cause of end-stage renal disease [1]. Thiamine and benfotiamine, a lipophilic thiamine-derivative, have been suggested as novel therapies for diabetic complications, including DN [2]. These agents would not exert their beneficial effects by improvement of hyperglycemia itself, but rather by activation of transketolase [2], [3]. This leads to a decrease in triosephospates and methylglyoxal; i.e. the major precursors of advanced glycation endproducts (AGEs), and subsequently inhibition of endothelial dysfunction and chronic low-grade inflammation [4], [5]. However, in a recent 12-week double-blind placebo-controlled trial in patients with type 2 diabetes, we found no effect of benfotiamine on urinary albumin excretion (UAE) or renal tubular damage markers [6]. One possibility is that our choice for these primary endpoints is too late in the sequence of events, because even the reduction in AGEs that occurs after pancreas transplantation has been reported to take years to translate into an effect on urinary albumin excretion [7]. We now aimed to evaluate the effect of benfotiamine on AGEs and markers of endothelial dysfunction and chronic low-grade inflammation, and to find ground to set up a study of longer duration.

## Methods

### Patients and study design

A detailed description of the study has been published [6]. The protocol for this trial and supporting CONSORT checklist are available as supporting information; see Protocol S1 and Checklist S1. We included patients from the outpatients department in the Isala Clinics, Zwolle, the Netherlands, in the period from January 2008 till June 2009. Included subjects were patients with type 2 diabetes, aged 40 to 75 years, with UAE between 15–300 mg/24h despite treatment with ACE inhibitors (ACE-Is) and/or angiotensin receptor blockers (ARBs). Patients (*n* = 86) were randomized to receive either benfotiamine (Wörwag pharma, Böblingen, Germany) 300 mg t.i.d. (total daily dose 900 mg) or placebo during 12 weeks. On each visit (baseline, 6 weeks, and 12 weeks), patients delivered 24-h urine collection, and additional morning spot-urine and blood samples were taken. All patients signed informed consent. This trial was conducted in accordance with the Helsinki Declaration and approved by the Medical Ethics Committee of the Isala Clinics, Zwolle, the Netherlands.

### Clinical laboratory investigations

For the current report, urine and plasma samples were stored frozen at −80°C until assessment. Detection of plasma AGEs *N* ^ε^-(carboxymethyl) lysine (CML), *N* ^ε^-(Carboxyethyl) lysine (CEL) and urine AGEs (CML, CEL and the methylglyoxal-arginine-adduct 5-hydro-5-methylimidazolone (MG-H1)) in 24-h urine samples was performed by means of a stable-isotope-dilution tandem mass spectrometry method [8], [9]. Soluble vascular cell adhesion molecule-1 (sVCAM-1), soluble intercellular adhesion molecule-1 (sICAM-1), high sensitivity C-reactive protein (hs-CRP) and serum amyloid-A (SAA) were assessed by multi-array detection system (SECTOR-Imager 2400, Mesoscale Discovery, Maryland). Soluble E-selectin and myeloperoxidase (MPO) were measured by commercially available multiplex assays (Millipore, Massachusetts). Other measurements were performed according to standard hospital procedures.

### Statistical analysis

Variables with a normal distribution are presented as mean and standard deviation and variables with a skewed distribution are presented as median and interquartile range. Intention-to-treat analysis and per-protocol analysis were planned. After randomization, four patients from the benfotiamine group withdrew from consent. Therefore, these subjects were not available for follow-up visits and no samples could be obtained from these subjects. All remaining patients (39 patients in the benfotiamine group and 43 patients in the placebo group) were available for analyses. In per-protocol analyses, patients who deviated from the study protocol (non-compliance or change in concomitant medications, *n* = 1 in the benfotiamine group and *n* = 3 in the placebo group [6]) were excluded. In the previous report [6], analyses of the primary endpoints (UAE and KIM-1) were presented. In this report, we present predefined analyses of secondary endpoints: plasma and urinary AGEs and plasma levels of biomarkers of endothelial dysfunction and low-grade inflammation. Using ANOVA for repeated measures (Mixed Model Analysis), within-subjects factors (effect of time; disease modifying model), effects of the between-subjects factors (difference between groups; symptomatic relief), and interaction between time of visit (0, 6, and 12 weeks) and group (benfotiamine versus placebo) were evaluated. Q-Q plots were used to assess whether the residuals of the dependent variables in the model had normal distribution. Because of skewed distribution of the residuals, logarithmic transformation (natural logarithm) of the data was performed before analysis. The results are summarized in terms of estimated means with 95% confidence intervals (CI). Differences in mean change between benfotiamine and placebo at 6 weeks and 12 weeks are presented as mean difference with 95% CI. *P*-values for time, group, and time*group interaction are presented. A two-sided *P*-value <0.05 was considered statistically significant. Statistics were done with SPSS, version 16.0 (Chicago, IL).

## Results

**Figure 1:**
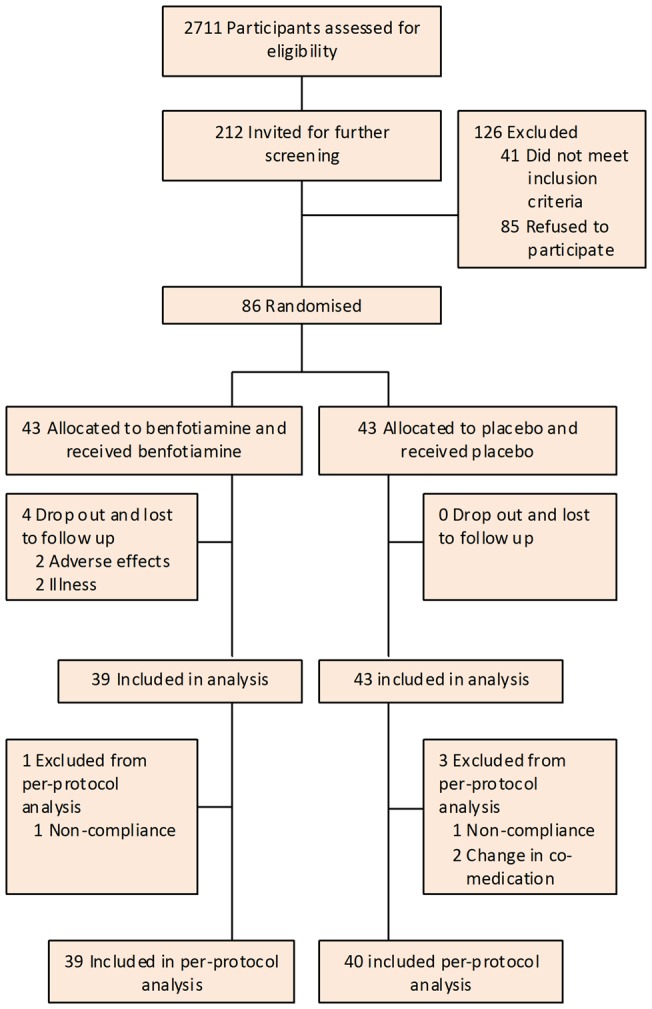
CONSORT flow diagram of the study.

A flow diagram of the trial is shown in figure 1. Baseline characteristics are presented in table 1. At baseline, blood thiamine was correlated with urinary excretion of CML (*r* = 0.26, *P* = 0.02) and CEL (*r* = 0.25, *P* = 0.02). No significant correlations of thiamine status with other AGEs or biomarkers of endothelial dysfunction and low-grade inflammation were found. Baseline characteristics were not materially different between groups, except for a more frequent use of oral hypoglycaemic agents and a slightly higher plasma creatinine in the placebo group. As shown in table 2, benfotiamine treatment had neither a significant effect on plasma or urinary AGEs nor on markers of endothelial dysfunction or chronic low-grade inflammation. Adjustment for baseline differences gave similar results. Results of per-protocol analysis (not shown) were not different from presented intention-to-treat analyses. Subgroup analyses in patients with low range UAE (<100 mg/24u) and high range UAE (>100 mg/24h) did not reveal differences in response to benfotiamine compared to placebo.

**Table 1:** Baseline characteristics of study population.

	Benfotiamine (*n* = 39)	Placebo (*n* = 43)
Males (*n* (%))	30 (77%)	33 (82%)
Age (years)	65.3 ± 5.9	64.6 ± 6.1
BMI (kg/m^2^)	32.1 ± 5.1	31.9 ± 5.9
Duration of diabetes (years)	12 [9; 18]	10 [7; 18]
SBP (mmHg)	140 ± 16	137 ± 20
DBP (mmHg)	67 ± 8	76 ± 10
A1c (%)	7.3 ± 0.9	7.4 ± 0.9
Plasma creatinine (μmol/l)	84 ± 19	87 ± 23
UAE (mg/24h)	90 [38; 267]	97 [48; 177]
Thiamine (nmol/l)	126 ± 23	122 ± 23
**Plasma AGEs**		
CML (nmol/mmol lysine)	64.48 [58.21; 69.69]	62.51 [54.88; 71.20]
CEL (nmol/mmol lysine)	51.14 [44.78; 59.25]	56.99 [43.71; 62.10]
**Urine AGEs**		
CML excretion (nmol/24h)	7630 [6761; 10576]	8879 [6476; 11769]
CML/creatinine (nmol/mmol)	572 [416; 731]	596 [483; 788]
CEL excretion (nmol/24h)	12405 [9105; 15240]	11204 [8922; 16384]
CEL/creatinine (nmol/mmol)	763 [602; 1061]	871 [648; 1034]
MG-H1 excretion (nmol/24h)	479122 [34431; 69775]	44930 [32095; 58614]
MG-H1/creatinine (nmol/mmol)	3459 [2196; 4856]	2999 [2260; 4563]
**Endothelial dysfunction markers**		
s-ICAM (ng/ml)	257.3 [222.0; 281.1]	241.7 [213.2; 308.0]
s-VCAM (ng/ml)	399.1 [362.7; 431.7]	388.3 [335.8; 461.9]
s-E-Selectin (ng/ml)	45.3 [29.8; 54.6]	39.5 [26.8; 51.3]
**Low-grade inflammation markers**		
Hs-CRP (ng/ml)	1395 [754; 2891]	1738 [824; 4097]
SAA (ng/ml)	1356 [927; 2028]	1162 [694; 2328]
MPO (ng/ml)	20.4 [9.9; 28.3]	20.4 [6.2; 27.2]

Data are mean ± standard deviation or median [interquartile range].

BMI, body mass index; SBP, systolic blood pressure; DBP, diastolic blood pressure; A1c, Glycated haemoglobin; UAE, urinary albumin excretion; AGEs, advanced glycation endproducts; CML, *N* ^ε^-(Carboxymethyl) lysine; CEL, *N* ^ε^-(Carboxyethyl) lysine; MG-H1, 5-hydro-5-methylimidazolone; s-ICAM, serum inter-cellular adhesion molecule-1; s-VCAM, serum vascular cell adhesion molecule-1; Hs-CRP, high sensitive C-reactive protein; SAA, serum amyloid A; MPO, myeloperoxidase.

**Table 2:** Estimated means of the log-transformed data at each visit, changes within group compared to baseline, and mean differences of change between groups compared to baseline.

	Benfotiamine (*n* = 39)	Placebo (*n* = 43)	Difference of change between groups	*P*		
				Time	Group	Time x Group
**Plasma AGEs**						
CML (nmol/mmol lysine)						
Baseline	4.17 (4.11 to 4.23)	4.15 (4.09 to 4.20)				
Week 6	4.15 (4.09 to 4.21)	4.14 (4.09 to 4.20)				
Week 12	4.15 (4.09 to 4.21)	4.12 (4.07 to 4.18)				
* At 6 weeks*	−0.02 (−0.12 to 0.08)	0.00 (−0.10 to 0.09)	−0.01 (−0.13 to 0.10)			
* At 12 weeks*	−0.02 (−0.12 to 0.08)	−0.02 (−0.12 to 0.07)	0.01 (−0.11 to 0.12)	0.77	0.50	0.95
CEL (nmol/mmol lysine)						
Baseline	3.94 (3.86 to 4.02)	3.97 (3.90 to 4.05)				
Week 6	3.88 (3.81 to 3.96)	3.94 (3.87 to 4.01)				
Week 12	3.90 (3.83 to 3.97)	3.98 (3.92 to 4.04)				
* At 6 weeks*	−0.06 (−0.18 to 0.07)	−0.03 (−0.16 to 0.09)	−0.02 (−0.17 to 0.12)			
* At 12 weeks*	−0.04 (−0.17 to 0.08)	0.01 (−0.11 to 0.13)	−0.05 (−0.19 to 0.09)	0.45	0.06	0.78
**Urine AGEs**						
U-CML excretion (nmol/24h)						
Baseline	8.98 (8.83 to 9.13)	9.08 (8.93 to 9.22)				
Week 6	8.99 (8.85 to 9.13)	9.08 (8.94 to 9.21)				
Week 12	9.04 (8.91 to 9.17)	9.02 (8.90 to 9.15)				
* At 6 weeks*	0.01 (−0.23 to 0.26)	0.00 (−0.23 to 0.24)	0.01 (−0.27 to 0.29)			
* At 12 weeks*	0.06 (−0.18 to 0.30)	−0.05 (−0.27 to 0.17)	0.11 (−0.16 to 0.38)	0.99	0.31	0.67
U-CML/creatinine (nmol/mmol)						
Baseline	6.29 (6.14 to 6.44)	6.40 (6.26 to 6.55)				
Week 6	6.30 (6.16 to 6.45)	6.31 (6.17 to 6.44)				
Week 12	6.30 (6.13 to 6.47)	6.38 (6.22 to 6.54)				
* At 6 weeks*	0.01 (−0.24 to 0.27)	−0.10 (−0.34 to 0.15)	0.11 (−0.18 to 0.40)			
* At 12 weeks*	0.01 (−0.27 to 0.29)	−0.02 (−0.28 to 0.24)	0.03 (−0.28 to 0.34)	0.82	0.29	0.74
U-CEL excretion (nmol/24h)						
Baseline	8.98 (8.83 to 9.13)	9.08 (8.93 to 9.22)				
Week 6	8.99 (8.85 to 9.13)	9.08 (8.94 to 9.21)				
Week 12	9.04 (8.91 to 9.17)	9.02 (8.90 to 9.15)				
* At 6 weeks*	0.01 (−0.23 to 0.26)	0.00 (−0.23 to 0.24)	0.01 (−0.27 to 0.29)			
* At 12 weeks*	0.06 (−0.18 to 0.30)	−0.05 (−0.28 to 0.18)	0.11 (−0.16 to 0.38)	0.99	0.31	0.67
U-CEL/creatinine (nmol/mmol)						
Baseline	6.29 (6.14 to 6.44)	6.40 (6.26 to 6.55)				
Week 6	6.30 (6.16 to 6.45)	6.31 (6.17 to 6.44)				
Week 12	6.30 (6.13 to 6.47)	6.38 (6.22 to 6.54)				
* At 6 weeks*	0.01 (−0.24 to 0.27)	−0.10 (−0.34 to 0.15)	0.11 (−0.18 to 0.40)			
* At 12 weeks*	0.01 (−0.27 to 0.29)	−0.02 (−0.28 to 0.24)	0.03 (−0.28 to 0.35)	0.82	0.29	0.74
MG-H1 excretion (nmol/24h)						
Baseline	10.74 (10.56 to 10.91)	10.72 (10.55 to 10.89)				
Week 6	10.63 (10.46 to 10.80)	10.72 (10.55 to 10.88)				
Week 12	10.63 (10.46 to 10.81)	10.58 (10.42 to 10.75)				
* At 6 weeks*	−0.10 (−0.40 to 0.19)	0.00 (−0.28 to 0.28)	−0.10 (−0.44 to 0.24)			
* At 12 weeks*	−0.10 (−0.40 to 0.20)	−0.14 (−0.42 to 0.15)	0.04 (−0.30 to 0.38)	0.38	0.94	0.69
U-MG-H1/creatinine (nmol/mmol)						
Baseline	8.05 (7.85 to 8.24)	8.05 (7.86 to 8.23)				
Week 6	7.94 (7.75 to 8.13)	7.94 (7.78 to 8.14)				
Week 12	7.90 (7.72 to 8.08)	7.90 (7.77 to 8.11)				
* At 6 weeks*	−0.10 (−0.44 to 0.23)	−0.08 (−0.40 to 0.23)	−0.02 (−0.40 to 0.36)			
* At 12 weeks*	−0.15 (−0.47 to 0.17)	−0.11 (−0.41 to 0.20)	−0.04 (−0.41 to 0.33)	0.36	0.80	0.98
**Endothelial dysfunction markers**						
s-ICAM (ng/ml)						
Baseline	5.53 (5.44 to 5.61)	5.55 (5.47 to 5.63)				
Week 6	5.55 (5.47 to 5.64)	5.53 (5.45 to 5.62)				
Week 12	5.56 (5.48 to 5.63)	5.52 (5.44 to 5.59)				
* At 6 weeks*	0.03 (−0.12 to 0.18)	−0.02 (−0.16 to 0.12)	0.05 (−0.12 to 0.22)			
* At 12 weeks*	0.03 (−0.11 to 0.17)	−0.03 (−0.17 to 0.10)	0.06 (−0.10 to 0.22)	0.99	0.74	0.74
s-VCAM (ng/ml)						
Baseline	5.98 (5.92 to 6.05)	5.98 (5.92 to 6.04)				
Week 6	5.99 (5.93 to 6.05)	5.96 (5.90 to 6.02)				
Week 12	6.02 (5.96 to 6.09)	5.96 (5.89 to 6.02)				
* At 6 weeks*	0.01 (−0.10 to 0.11)	−0.02 (−0.12 to 0.09)	0.02 (−0.10 to 0.14)			
* At 12 weeks*	0.04 (−0.07 to 0.15)	−0.02 (−0.12 to 0.08)	0.06 (−0.07 to 0.18)	0.91	0.19	0.64
s-E-selectin (ng/ml)						
Baseline	3.66 (3.46 to 3.85)	3.57 (3.39 to 3.75)				
Week 6	3.84 (3.70 to 3.97)	3.71 (3.59 to 3.84)				
Week 12	3.79 (3.64 to 3.95)	3.69 (3.54 to 3.84)				
* At 6 weeks*	0.18 (−0.10 to 0.46)	0.14 (−0.13 to 0.41)	0.04 (−0.28 to 0.37)			
* At 12 weeks*	0.14 (−0.16 to 0.44)	0.12 (−0.17 to 0.40)	0.02 (−0.32 to 0.36)	0.14	0.10	0.96
**Low-grade inflammation markers**						
Hs-CRP (ng/ml)						
Baseline	7.49 (7.13 to 7.85)	7.56 (7.21 to 7.91)				
Week 6	7.63 (7.28 to 7.98)	7.41 (7.07 to 7.75)				
Week 12	7.54 (7.20 to 7.87)	7.51 (7.19 to 7.83)				
* At 6 weeks*	0.14 (−0.48 to 0.75)	−0.15 (−0.74 to 0.44)	0.29 (−0.41 to 0.98)			
* At 12 weeks*	0.05 (−0.55 to 0.64)	−0.05 (−0.63 to 0.52)	0.10 (−0.59 to 0.78)	0.99	0.67	0.71
SAA (ng/ml)						
Baseline	7.26 (6.95 to 7.58)	7.22 (6.92 to 7.53)				
Week 6	7.29 (6.96 to7.61)	7.07 (6.76 to 7.38)				
Week 12	7.14 (6.83 to 7.46)	7.20 (6.90 to 7.50)				
* At 6 weeks*	0.02 (−0.53 to 0.57)	−0.15 (−0.68 to 0.37)	0.18 (−0.45 to 0.81)			
* At 12 weeks*	−0.12 (−0.66 to 0.42)	−0.02 (−0.54 to 0.50)	−0.10 (−0.72 to 0.51)	0.88	0.60	0.67
MPO (ng/ml)						
Baseline	2.90 (2.61 to 3.20)	2.80 (2.51 to 3.09)				
Week 6	2.89 (2.61 to 3.17)	2.67 (2.40 to 2.94)				
Week 12	2.88 (2.61 to 3.16)	2.72 (2.46 to 2.98)				
* At 6 weeks*	−0.02 (−0.52 to 0.48)	−0.13 (−0.61 to 0.35)	0.11 (−0.45 to 0.68)			
* At 12 weeks*	−0.02 (−0.51 to 0.47)	−0.08 (−0.56 to 0.39)	0.06 (−0.50 to 0.63)	0.87	0.15	0.92

Data are log-transformed and presented as mean (95% confidence interval) or mean difference (95% confidence interval for difference).

CML, *N* ^ε mn^-(Carboxymethyl) lysine; CEL, *N* ^ε^-(Carboxyethyl) lysine; MG-H1, 5-hydro-5-methylimidazolone; s-ICAM, serum inter-cellular adhesion molecule-1; s-VCAM, serum vascular cell adhesion molecule-1; Hs-CRP, high sensitive C-reactive protein; SAA, serum amyloid A; MPO, myeloperoxidase.

## Discussion

We found that 12-week treatment with benfotiamine in patients with type 2 diabetes did not result in a decrease of plasma AGEs, urinary excretion of AGEs, plasma biomarkers of endothelial dysfunction or plasma biomarkers of chronic low-grade inflammation.

Benfotiamine is converted to thiamine pyrophosphate, a co-factor of transketolase. The activation of transketolase plays a crucial role in oxidative and non-oxidative pentosephosphate pathways that inhibit vascular complications of diabetes [3], [10]. In our trial, although we found that benfotiamine resulted in a significant improvement in thiamine status (a significant increase in thiamine levels and transketolase activity), there was no significant difference between the benfotiamine group and the placebo group regarding urinary albumin excretion, urinary excretion of tubular damage markers, or blood pressure [6].

Studies in diabetic animals have shown that benfotiamine inhibited protein kinase C, formation of AGEs and oxidative stress, indicating a protective effect against hyperglycemia-induced endothelial dysfunction and inflammation [2], [4].

To the best of our knowledge, our study is the first randomised controlled trial that has investigated the effect of benfotiamine on AGEs formation and markers of low-grade inflammation in humans. Results from previous studies on thiamine and benfotiamine on endothelial function in humans are conflicting. An earlier cross-sectional analysis in 74 patients with type 1 and type 2 diabetes found an inverse association of thiamine status with sVCAM-1 [11], suggesting an effect of thiamine status on markers of endothelial dysfunction. However, in a study in patients with type 2 diabetes [5], the same group found no effect of thiamine supplementation on sVCAM-1, We also did not find an effect of benfotiamine on markers of endothelial dysfunction, including sVCAM-1. Nevertheless, in a study in 13 patients with type 2 diabetes [12], beneficial effects of benfotiamine (1050 mg/day) on the endothelial damage and oxidative stress that occurs after consumption of an AGE-rich meal were suggested to be already present after three days of treatment. While on the basis of this experiment longer periods of treatment with benfotiamine (12 weeks in our study) might be anticipated to be sufficient to cause an effect on formation of AGEs and endothelial damage, the effects of benfotiamine could also be more directly meal-related rather than affecting steady-state concentrations.

Compared to previous studies in animals [2] and humans [5], [13], a daily dose of 900 mg is considered as a high dose. Due to its pharmacokinetics, benfotiamine is absorbed considerably better than thiamine. However, it is important to realize that intestinal and renal tubular transport of thiamine is tightly regulated to maintain homeostasis. Accordingly, expression of the transporters is up-regulated in presence of deficiency states and down-regulated in response to an excess supply of these nutrients, which may account for a rapid excretion of thiamine in the urine after consumption of a high dose of benfotiamine [14], [15]. Additionally, studies in experimental animals have shown that chronic kidney disease results in down-regulation of expression of key transporters and receptors for thiamine and folate, which can further limit the bioavailability of these micronutrients [16]. In our study, although higher blood thiamine levels and transketolase activity was achieved in the benfotiamine group, thiamine levels in renal cells might still be deficient even in case of high blood thiamine.

Our objectives were studied in a relevant study population, including patients with type 2 diabetes and UAE in the high-normal and microalbuminuric range treated with ACE-Is and/or ARBs. An important limitation of this study is that AGEs and biomarkers of endothelial dysfunction and inflammation were measured in urine and blood. Intracellular concentrations of these markers in target organs (e.g. glomeruli and tubuli) may give additional information on effects of benfotiamine on vascular complications. Therefore, assessment of intracellular AGEs in future studies would be necessary.

In conclusion, 12-week benfotiamine treatment did neither significantly affect plasma or urine AGEs nor plasma biomarkers of endothelial dysfunction or chronic low-grade inflammation in patients with type 2 diabetes and UAE in the high-normal and microalbuminuric range. Because small short-term effects can not be excluded and potential effects that take long to ensue can not be studied in a 12-week study, larger or longer term studies are necessary to elucidate whether benfotiamine reduces formation of AGEs or diminishes the risk of hyperglycemia-induced vascular complications.

## Supporting Information

Protocol S1 **Trial protocol.**
(DOC)

Checklist S1 **CONSORT checklist of the trial.**
(DOC)
