# Supplementary material for: Effect of Benfotiamine on Advanced Glycation Endproducts and Markers of Endothelial Dysfunction and Inflammation in Diabetic Nephropathy
Source: PLoS One. 2012 Jul 6;7(7):e40427. doi: 10.1371/journal.pone.0040427 (PMC3391239; doi:10.1371/journal.pone.0040427)
Supplement: Protocol S1 — Trial protocol. (DOC) [file pone.0040427.s001.doc]

***‘A Double-Blind Clinical Trial of Benfotiamine Treatment in Diabetic Nephropathy’***

| **Protocol ID** | ***BENFO-1*** |
| --- | --- |
| **Short title** | ***Benfotiamine in Diabetic Nephropathy*** |
| **Version** | **3.0** |
| **Date** | **07/04/2008** |
| **Coordinating investigator/project leader** | ***University Medical Center Groningen, UMCG***  ***Prof. Dr. G.J. Navis, internist-nephrologist***  ***Postbus 30.001, 9700 RB Groningen***  ***Tel: 050 361 4441***  ***E-mail: g.j.navis@int.umcg.nl*** |
| **Principal investigator** | ***Isala Klinieken, Locatie Sophia***  ***Prof. Dr. H.J.G. Bilo, internist-nephrologist***  ***Postbus 10400, 8000 GK Zwolle***  ***Tel: 038 424 2518***  ***E-mail: h.j.g.bilo@isala.nl*** |
| **Researcher** | ***A. Alkhalaf, research fellow***  ***Isala klinieken, locatie Sophia***  ***Postbus 10400, 8000 GK Zwolle***  ***Tel: 038 424 7942***  ***E-mail: a.alkhalaf@isala.nl*** |
| **Sponsor** | ***University Medical Center Groningen, UMCG***  ***Dr. S.J.L Bakker, internist-nephrologist***  ***Postbus 30.001, 9700 RB Groningen***  ***Tel: 050 361 3677***  ***Email: s.j.l.bakker@int.umcg.nl*** |
| **Independent physicians** | ***- Dr. S.H.A Diepeveen, internist-nephrologist***  ***Isala Klinieken, Locatie Sophia***  ***Tel: 038 424 2275***  ***E-mail: s.h.a.diepeveen@isala.nl*** |
|  | ***- Dr. M. Bijl, internist- rheumatologist***  ***University Medical Center Groningen, UMCG***  ***Tel: 050 361 2903***  ***E-mail: m.bijl@int.umcg.nl*** |
| **Laboratory sites** | ***- Chemical Medical laboratory***  ***Isala Klinieken, locatie Sophia,***  ***Tel: 038 424 24 68*** |
|  | ***- Central Clinical Laboratory***  ***University Medical Center Groningen***  ***Tel:*** ***050 361 61 61*** |
| **Pharmacy** | ***- Isala Klinieken pharmacy***  ***Isala Klinieken, locatie Sophia,***  ***Tel: 038 424 24 69*** |

**PROTOCOL SIGNATURE SHEET**

| **Name** | **Signature** | **Date** |
| --- | --- | --- |
| **Prof. Dr. R.O.B. Gans**,  **Internal Medicine, Department Head**  **University Medical Center Groningen** |  |  |
| **Prof. Dr. H.J.G Bilo, internist**  **Isala Klinieken Locatie Sophia,**  **and University Medical Center Groningen** |  |  |

**TABLE OF CONTENTS**

1. INTRODUCTION AND RATIONALE [10](#__RefHeading___Toc176794667)

2. OBJECTIVES [11](#__RefHeading___Toc176794668)

3. STUDY DESIGN [11](#__RefHeading___Toc176794669)

4. STUDY POPULATION [12](#__RefHeading___Toc176794670)

4.1 Population [12](#__RefHeading___Toc176794671)

4.2 Inclusion criteria [12](#__RefHeading___Toc176794672)

4.3 Exclusion criteria [13](#__RefHeading___Toc176794673)

4.4 Sample size calculation [14](#__RefHeading___Toc176794674)

5. TREATMENT OF SUBJECTS [14](#__RefHeading___Toc176794675)

5.1 Investigational product/treatment [14](#__RefHeading___Toc176794676)

5.2 Use of co-intervention [14](#__RefHeading___Toc176794677)

6. INVESTIGATIONAL MEDICINAL PRODUCT [15](#__RefHeading___Toc176794678)

6.1 Name and description of investigational medicinal product [15](#__RefHeading___Toc176794679)

6.2 Summary of findings from non-clinical studies [15](#__RefHeading___Toc176794680)

6.3 Summary of findings from clinical studies [15](#__RefHeading___Toc176794681)

6.4 Summary of known and potential risks and benefits [15](#__RefHeading___Toc176794682)

6.5 Description and justification of route of administration and dosage [15](#__RefHeading___Toc176794683)

6.6 Dosages, dosage modifications and method of administration [16](#__RefHeading___Toc176794684)

6.7 Preparation and labelling of Investigational Medicinal Product [17](#__RefHeading___Toc176794685)

6.8 Drug accountability [17](#__RefHeading___Toc176794686)

7. METHODS [17](#__RefHeading___Toc176794687)

7.1 Study parameters/endpoints [17](#__RefHeading___Toc176794688)

7.1.1 Main study parameter/endpoint [17](#__RefHeading___Toc176794689)

7.1.2 Secondary study parameters/endpoints [17](#__RefHeading___Toc176794690)

7.1.3 Other study parameters [18](#__RefHeading___Toc176794691)

7.2 Randomisation, blinding and treatment allocation [18](#__RefHeading___Toc176794692)

7.3 Study procedures [19](#__RefHeading___Toc176794693)

7.4 Withdrawal of individual subjects [19](#__RefHeading___Toc176794694)

7.4.1 Specific criteria for withdrawal [19](#__RefHeading___Toc176794695)

7.5 Replacement of individual subjects after withdrawal [20](#__RefHeading___Toc176794696)

7.6 Follow-up of subjects withdrawn from treatment [20](#__RefHeading___Toc176794697)

7.7 Premature termination of the study [20](#__RefHeading___Toc176794698)

8. SAFETY REPORTING [20](#__RefHeading___Toc176794699)

8.1 Section 10 WMO event [20](#__RefHeading___Toc176794700)

8.2 Adverse and serious adverse events [21](#__RefHeading___Toc176794701)

8.2.1 Suspected unexpected serious adverse reactions (SUSAR) [21](#__RefHeading___Toc176794702)

8.2.2 Annual safety report [22](#__RefHeading___Toc176794703)

8.3 Follow-up of adverse events [22](#__RefHeading___Toc176794704)

9. STATISTICAL ANALYSIS [23](#__RefHeading___Toc176794705)

9.1 Descriptive statistics [23](#__RefHeading___Toc176794706)

9.2 Confirmatory testing [23](#__RefHeading___Toc176794707)

9.3 Explorative analyses [23](#__RefHeading___Toc176794708)

10. ETHICAL CONSIDERATIONS [23](#__RefHeading___Toc176794709)

10.1 Regulation statement [23](#__RefHeading___Toc176794710)

10.2 Recruitment and consent [24](#__RefHeading___Toc176794711)

10.3 Benefits and risks assessment, group relatedness [24](#__RefHeading___Toc176794712)

10.4 Compensation for injury [25](#__RefHeading___Toc176794713)

10.5 Incentives [25](#__RefHeading___Toc176794714)

11. ADMINISTRATIVE ASPECTS AND PUBLICATION [25](#__RefHeading___Toc176794715)

11.1 Handling and storage of data and documents [26](#__RefHeading___Toc176794716)

11.2 Amendments [26](#__RefHeading___Toc176794717)

11.3 Quality control and Quality Assurance [26](#__RefHeading___Toc176794718)

11.4 Annual progress report [27](#__RefHeading___Toc176794719)

11.5 End of study report [27](#__RefHeading___Toc176794720)

11.6 Public disclosure and publication policy [27](#__RefHeading___Toc176794721)

12. Reference List [29](#__RefHeading___Toc176794722)

**LIST OF ABBREVIATIONS AND RELEVANT DEFINITIONS**

| **ABR** | **ABR form (General Assessment and Registration form) is the application form that is required for submission to the accredited Ethics Committee (ABR = Algemene Beoordeling en Registratie)** |
| --- | --- |
| **AE** | **Adverse Event** |
| **AR** | **Adverse Reaction** |
| **CA** | **Competent Authority** |
| **CCMO** | **Central Committee on Research Involving Human Subjects** |
| **CV** | **Curriculum Vitae** |
| **DSMB** | **Data Safety Monitoring Board** |
| **EU** | **European Union** |
| **EudraCT** | **European drug regulatory affairs Clinical Trials GCP Good Clinical Practice** |
| **IB** | **Investigator’s Brochure** |
| **IC** | **Informed Consent** |
| **IMP** | **Investigational Medicinal Product** |
| **IMPD** | **Investigational Medicinal Product Dossier** |
| **METC** | **Medical research ethics committee (MREC); in Dutch: medisch ethische toetsing commissie (METC)** |
| **(S)AE** | **Serious Adverse Event** |
| **SPC** | **Summary of Product Characteristics (in Dutch: officiële productinfomatie IB1-tekst)** |
| **Sponsor** | **The sponsor is the party that commissions the organisation or performance of the research, for example a pharmaceutical**  **company, academic hospital, scientific organisation or investigator. A party that provides funding for a study but does not commission it is not regarded as the sponsor, but referred to as a subsidising party.** |
| **SUSAR** | **Suspected Unexpected Serious Adverse Reaction** |
| **Wbp** | **Personal Data Protection Act (in Dutch: Wet Bescherming Persoonsgevens)** |
| **WMO** | **Medical Research Involving Human Subjects Act (Wet Medisch-wetenschappelijk Onderzoek met Mensen** |

**SUMMARY**

**Rationale:** Diabetic nephropathy is a serious complication of diabetes mellitus, which is the leading cause of end-stage renal disease (ESRD) in the Western World. Benfotiamine has been shown to reduce diabetic nephropathy and retinopathy in animal experimental models. We will investigate the effect of benfotiamine supplementation in patients with diabetic nephropathy, and hypothesize that it will slow down the progression to ESRD if it leads to reduction of tubulointerstitial damage and inflammation.

**Objectives**:

-To investigate whether short-term treatment (3 months) with benfotiamine leads to a reduction in urinary excretion of β2-microglobulin and albumin.

-To investigate whether short-term treatment (3 months) with benfotiamine leads to reduction in urinary excretion of markers of renal tubulointerstitial damage and inflammation (Kidney Injury Molecule-1, Monocyte Chemo-attractant Protein-1, Macrophage Inhibiting Factor, Complement factor C3d)

**Study design:** Mono-center, randomized, controlled, double-blind, parallel group clinical trial studying the effect of high dose benfotiamine compared to placebo in 86 patients with diabetic nephropathy. The study consists of 2 phases;

1-Run-in phase (6 weeks): In this phase patients will be instructed on collection of 24h urine samples, it will be checked whether they have urinary albumin excretion consistently in the microalbuminuric range, and they will be instructed on maintenance of stable diet and activity levels prior to the study visits to the outpatient clinic.

2-Treatment phase (12 weeks): According to randomization, benfotiamine is given to one half of the patients and placebo is given to the other half.

**Study population:** ***40–75 year old patients with type 2 diabetes mellitus (T2DM)*** and diabetic nephropathy with microalbuminuria (30-300 mg/24 h urine) and glomerular filtration rate (eGFR, estimated by MDRD formula) > 30 ml/min

**Intervention**
Group A: Benfotiamine (300 mg) 3x 1 film coated tablet daily (900 mg daily dose benfotiamine)
Group B: Placebo 3x 1 film coated tablet daily

The intervention duration is 12 weeks for each group.

**Main study parameters/endpoints:**

Change in urinary excretion of:

• β2-microglobulin

• Albumin

**Nature and extent of the burden and risks associated with participation, benefit and group relatedness:**

-The patients are asked to attend the outpatient clinic for the study 5 times in total. Before each visit the patients are asked to collect a 24h urine sample. They will be asked to refrain from food from the previous evening to take fasting blood samples (5 vials of 7 ml). In addition, a second-morning urine sample will be collected.

- No other physical or physiological discomfort in association with participation is anticipated than the discomfort associated with the collection of the 24h urine samples and the blood sampling. There are no known risks associated with the investigational product other than the rare occurrence of urticaria and pruritus. In case of positive effects on diabetic nephropathy (albuminuria) a program on prophylaxis and treatment of diabetic nephropathy with benfotiamine will be taken into account. Benfotiamine will be investigated to assess its effect on markers of tubulointerstitial damage and inflammation associated with diabetic nephropathy. If it will be demonstrated that benfotiamine causes an improvement in these markers, the drug may become eligible for further large studies on its efficacy in prevention of the development of end-stage renal disease in susceptible diabetic patients. Participation of diabetic nephropathy patients in this study is of great importance, and answering the questions of this clinical trial is not possible without observing and analyzing the parameters at the end of the study.

# INTRODUCTION AND RATIONALE

There is a worldwide increase in prevalence in type 2 diabetes mellitus (1), which is being paralleled by an increasing number of patients reaching dialysis because of diabetic nephropathy (2). Much of the fivefold increase in patients receiving dialysis treatment that occurred over the past two decades is attributable to type 2 diabetes and diabetic nephropathy (3). Diabetes is now the leading cause of end-stage renal disease (ESRD), with more than 40% of all new cases of ESRD occurring in patients with diabetes (3).

The pathophysiology of diabetic nephropathy and progression to ESRD is essentially composed of two components. The first is induction of glomerular endothelial dysfunction by hyperglycaemia, leading to albuminuria (4). The second is tubulo-interstitial inflammation and fibrosis, which is much more the consequence of albuminuria (5,6), although hyperglycaemia may accelerate the process (7). Tight glycaemic control has been shown to reduce the risk for development of albuminuria in patients with diabetes, as shown in several controlled trials in type 1 and type 2 diabetes (8-11). However, once established, glomerular lesions and albuminuria are reversible, but poorly. It has been shown to take at least five years of normalisation of glucose concentrations by pancreas transplantation before glomerular lesions and albuminuria decreased (12). Glycaemic control has, therefore, only limited influence on progression to ESRD once albuminuria is present (2). Currently, reduction of albuminuria with antihypertensive/antiproteinuric treatment, in particular with angiotensin-converting enzyme inhibitors (ACEi) and angiotensin II antagonists (AIIA) is the cornerstone of prevention of progression of overt diabetic nephropathy to ESRD (13-15). Reductions in relative risk for decline in renal function were approximately 50% with ACEi treatment in patients with type 1 diabetes (13). These risk reductions were considerably less – approximately 20% – for the end-point of doubling of serum creatinine or reaching ESRD in type 2 diabetic patients with diabetic nephropathy (14,15).

Thus, there is great need for further adjunctive treatments that can prevent progressive decline in renal function in patients with diabetic nephropathy, in type 2 diabetes in particular. Several studies suggest that in non-diabetic proteinuric diseases, albuminuria and/or proteinuria induce oxidative stress and accumulation of advanced glycation end-products (AGEs) (independent of glucose concentrations) in tubular epithelial cells and the renal interstitium, which elicit proinflammatory and profibrotic responses that contribute to chronic tubulointerstitial damage and loss of renal function (16-21). Hyperglycaemia, which is also known to induce oxidative stress and AGE accumulation, may obviously enhance these detrimental effects of albuminuria/proteinuria (7). The lipid-soluble thiamine derivative benfotiamine, has recently been shown to be able to oppose the induction of oxidative stress and AGE accumulation by hyperglycaemia (22). Optimal thiamine availability is crucial for the maintenance of enzymatic anti-oxidant defence in cells (23,24). Benfotiamine has recently been shown to be able to reduce diabetic nephropathy and retinopathy in animal experimental models (22,25). Thornalley et al have recently found that diabetes induces thiamine deficiency in tissues in humans (26). We therefore hypothesize that benfotiamine supplementation in patients with diabetic nephropathy will ameliorate the effects of both albuminuria/proteinuria and hyperglycaemia on oxidative stress and AGE accumulation in renal tissue, and thereby decrease inflammatory and fibrotic responses, causing slowing down of progression to ESRD as a consequence.

# OBJECTIVES

*Primary Objectives:*

-Does short-term treatment (3 months) with benfotiamine in diabetic nephropathy result in a lowering of urinary excretion of β2-microglobulin and albumin?

*Secondary Objectives:*

-Does short-term treatment (3 months) with benfotiamine result in a lowering of urinary excretion of established and emerging markers of tubulointerstitial damage and inflammation, such as KIM-1, MIF, MCP-1, and C3d in patients with diabetic nephropathy (the expression of MCP-1 in the kidney and the amounts of MCP-1 in urine have, for instance, been shown to be associated with severity of tubulointerstitial disease and to predict the loss of renal function in several types of chronic kidney disease (27-32))?

# STUDY DESIGN

In this mono-center, randomized, controlled, double-blind clinical trial with 2 parallel groups (verum versus placebo) to investigate the effect of high dose benfotiamine on urinary excreation of β2-microglobulin and albumin in patients with diabetic nephropathy. The study medication will be added upon existing treatment for diabetic nephropathy. The study consists of following phases:

***Run-in phase (6 weeks):***

During this phase, patients will be instructed on collection of 24-hour urine sample before each visit to the outpatient clinic. Patient will also be checked whether they have urinary albumin excretion consistently in the microalbuminuric range by doing a 24 hour-urine analysis on two different occasions with 2-6 weeks in between. They will be instructed on maintenance of stable diet and physical activity prior to each visit to the outpatient clinic.

***Treatment phase (12 weeks):***

After the patients are randomly divided into two groups, the treatment is given for both groups for 12 weeks in a double-blind pattern. One group will be given the study medication, and the other will be given the placebo.

During this phase patient are asked to attend the outpatient clinic 2 times:

*-Week 0 (the beginning of the phase)*

*-Week 6*

*-Week 12 (the end of the phase)*

In each visit of these, fasting blood samples (5 vials of 7 ml) are drawn for analysis (blood glucose, HBA1c, and other parameters), second-morning urine sample is collected, and 24-hour urine (of the preceding day) is sent to the laboratory for analysis.

# STUDY POPULATION

## Population

The population of this trial consists of 40-75 year old, male and female patients with type 2 diabetes mellitus.

## Inclusion criteria

• Type 2 diabetes mellitus
• Age: 40-75 years
• Patients are on treatment with angiotensin converting enzyme inhibitors (ACEi) and/or angiotensin II antagonists (AIIA) in an unchanged dose for at least 3 months
• Active diabetic nephropathy as indicated by presence of microalbuminuria (30-300 mg/24 h urine) in at least two samples within 2-6 weeks in advance of inclusion in the trial
• HbA1c < 8.5%, a higher HbA1c ≤ 9.5% is acceptable if the treating physician and the patient have accepted that striving for lower values is an unreachable goal (patients with high HbA1c values are the ones that one would expect to be benefit most from treatment with benfotiamine)
• eGFR (estimated by MDRD formula) > 30 ml/min
• Males and postmenopausal females
• Written informed consent

## Exclusion criteria

• Renal impairment by other causes than diabetes
• Stage of the disease more severe than indicated in "Inclusion criteria"
(macroalbuminuria or renal insufficiency)
• Severe hypoglycemia during the last 3 months, needing help from another person
• Severe hepatopathy (liver function enzymes about three times higher than normal)
• Endocrine disorders, e.g. hyper-/hypothyroidism
• Blood pressure > 160/90 mmHg
• Severe cardiac function disturbances and severe heart rhythm disturbances
• Neoplasm
• Severe general diseases or mental disorders making the participation in the study
impossible
• Drug abuse
• Female patients during pregnancy and lactation period and female patients with active menses during the past year
• Hypersensitivity to benfotiamine or other constituents of the study medication
• HbA1c >9.5%
• Use of vitamin B containing supplements during the last 3 months
• Use of NSAIDs more than 3x per week (including self-medication)

• Participation in another study within one month before joining the benfotiamine
study

## Sample size calculation

The two primary outcome variables are quantitative. As primary outcome measure, the difference between the value at the end and the value at the start of medication is defined. It is assumed that the differences are normally distributed. P-values are adjusted for multiple testing by the Bonferroni-Holm-procedure. Hypotheses are one sided. As level of significance 2.5% are established.

It is proposed to have 38 evaluable patients per group. This sample size enables the study to detect an effect of size 0.65 with a power of 80%. Effect size is the difference in group means divided by the common standard deviation. For clarification: If (mean of the placebo group minus the mean of the verum group ) divided by the standard deviation is bigger or equal 0.65 then the study will deliver a significant result with a probability of at least 80%.

The drop-out rate is assumed to be 10%. Therefore 43 patients have to be randomised to each group to have 38 evaluable patients. For both groups together 86 patients are needed for admission to the study.

# TREATMENT OF SUBJECTS

## Investigational product/treatment

Benfotiamine is the investigational treatment in this clinical trial. The medicinal product benfotiamine is a lipid-soluble derivative of thiamine (vitamin B1). The effect of this treatment will be compared to placebo in the study population.

## Use of co-intervention

During the run-in phase, treatment of the patients will be optimized in order to keep it mostly constant during the study period. Special attention has to be payed for following concomitant treatments which must not be changed during study:

- ACE inhibitors / RAS blockers
- Acetylic salicylic acid / NSAIDs
- Metformin
- Statins

If one of these medications has to be changed substantially during the study, the patient can continue the study or has to be withdrawn. In case of continuation of the study, the changes of medication have to be documented thoroughly.

# INVESTIGATIONAL MEDICINAL PRODUCT

## Name and description of investigational medicinal product

*Milgamma® mono 300*

Film coated tablets

Each tablet contains 300 mg benfotiamine

*Placebo*:

Film coated tablets

Each tablet contains microcrystalline cellulose instead of benfotiamine

## Summary of findings from non-clinical studies

Please see attached Summary of Product Characteristics, pages 2, 3

## Summary of findings from clinical studies

Pleae see attached Summary of Product Characteristics, pages 2, 3

## Summary of known and potential risks and benefits

Please see attached Summary of Product Characteristics, pages 2, 3, 4

## Description and justification of route of administration and dosage

The dose of the active study medication, benfotiamine, is chosen according to known clinical and experimental data.

For the treatment of diabetic polyneuropathy, daily doses up to 600 mg alone (33,35) or 320 mg in combination with pyridoxin (720 mg) and cyanocobalamine (2 mg) have been used (36-38).

The treatment of patients with type-II diabetes with 600 mg benfotiamine per day for 4 weeks leads to a reduction of CML (N-carboxmethyl-Lysin) in erythrocytes, a marker of intracellular oxidative stress (39). Similar results in patients with type-I diabetes have been shown in an open study too (40). Stirban et al. (41,42) used daily doses of 3x350 mg (1.050 mg) for 3 days to show that this dose significantly decreased vascular impairment after an AGE-rich meal in patients with type-II diabetes.

In diabetic rats, high oral doses of benfotiamine (7mg and 70mg per kg body weight) prevented the development of microalbuminuria by 70-80% (25). Furthermore, in another trial retinopathy in diabetic rats was prevented by high-dose benfotiamine (80 mg/kg body weight daily) (22).

Generally, it is accepted that lipophilic as well as water soluble thiamine derivatives have a very low toxicity and excellent tolerability. According to case reports, toxic effects of thiamine may occur after oral application of >3 g daily (43), which is higher than normal experimental or therapeutic dosage. Other case reports describe symptoms of a hyperthyroidism after doses of thiamine hydrochloride of ≥7g or 10g over 2 weeks (44,45). After discontinuation of thiamine hydrochloride, the symptoms resolved within 2 days. For benfotiamine no reports on overdose are known. In all clinical studies with benfotiamine performed up to now no serious adverse events have been observed. Only in single cases an allergic reaction may be possible. Therefore, patients with hypersensitivity to benfotiamine (or other constituents of the study medication, see SPC) should be excluded from the study.

## Dosages, dosage modifications and method of administration

*Dose and application interval:*

Treatment phase 1:

Group A: milgamma® mono 300 3x 1 film coated tablet daily (total 900 mg daily dose benfotiamine)

Group B: Placebo 3x 1 film coated tablet daily

*Route of administration:* Oral use

The pills should be swallowed without chewing with some fluid in the morning, at noon and in the evening. The intake is independent from meals.

## Preparation and labelling of Investigational Medicinal Product

Study medication containing benfotiamine corresponds to the approved drug milgamma® mono 300 (see SPC). Placebo is produced by the same manufacturer replacing benfotiamine by one of the other excipients (microcristalline cellulose).

For labelling of the study medication, see Attachment “Labelling”

## Drug accountability

The study medication is packed according to the randomization list by the manufacturer. For each patient, per treatment period an amount of 3x 100 film coated tablets are prepared. Each package contains 10 blisters of 10 film coated tablets.

After approval by the QP of the manufacturer, it is shipped to the clinic pharmacy of Isala Klinieken, Locatie Sophia. The pharmacy dispends the study medication to the investigator on request, who distributes the study medication to the patients.

After any treatment period, patients are asked to return the medication not taken or the empty package to the investigator. Rest medication is collected in the pharmacy of the clinic and sent back to Wörwag Pharma GmbH & Co. KG after end of the trial, where it is professionally eliminated.

# METHODS

## Study parameters/endpoints

### Main study parameter/endpoint

Change in urinary excretion of (measured in 24-hour urine):

• β2-microglobulin

• Albumin

### Secondary study parameters/endpoints

Change in urinary excretion of (measured in 24-hour urine):

• KIM-1 (kidney injury molecule-1)
• MIF (macrophage inhibiting factor)
• MCP-1 (monocyte chemo-attractant protein-1)
• C3d
• Urinary peptidomics
• Reactive oxygen species, advanced glycation end-products (AGEs), and carbonylated albumin

### Other study parameters

• Body Mass Index (BMI)

• Blood glucose

• HbA1c

• Blood urea nitrogen

• Plasma creatinine

• Blood thiamine

• Erythrocyte transketolase activity

## Randomisation, blinding and treatment allocation

The treatment phases of 3 months is performed in a double-blind design. Active medication and placebo have identical appearance and can be identified within this study only by the randomizing list.

A randomizing list will be prepared by Prof. Dr. W. Gaus (independent statistician, University of Ulm, Germany) and sent to the manufacturer of the study medication for packaging. The randomizing list is to be stored strictly closed and must not be seen by anyone else than the packaging employee. After packaging, it has to be sealed and sent to Wörwag Pharma GmbH & Co. KG, where it is stored safe and strictly closed. A duplicate of the randomization list will also be stored in a safe by the statistician Prof. Dr. W. Gaus for safety reasons, if the original list gets lost. After correct data unblinding by the original list, this duplicate list will be eliminated.

Additionally to the randomizing list for packaging of the study medication, two sets of sealed envelopes are prepared by the statistician for emergency purposes. Each envelope contains the allocation to the study medication for an individual patient. One set of sealed envelopes will be sent to the investigator, one set will be safely stored at Wörwag Pharma GmbH & Co. KG. If a serious adverse event occurs that requires the unblinding of a single patient, this will be performed by opening the envelope of this patient.

The reason for and the date of unblinding has to be documented on the envelope and in the CRF. All other patients remain blinded.

After the end of the study, all envelopes of the investigator have to be sent to the biometric centre. The integrity of the envelopes has to be documented by a third party.

## Study procedures

The patients will be instructed in advance how to collect the 24-hour urine in the previous day, and will be asked to refrain from food from the previous evening to take fasting blood samples

The subjects will visit the clinic 5 times during this clinical trial. Two times in the run in phase, one time directly before the beginning of the treatment phase, one time in the middle of the treatment phase, and one last time at the end of the treatment phase. They will be instructed in advance how to collect the 24-hour urine in the previous day, and will be asked to refrain from food from the previous evening to take fasting blood samples

In each visit the following procedures will be applied:

- Samples of 24-hour urine will be received from the patients and sent to the laboratory for analysis. The primary and secondary study parameters will be measured in the 24-hour urine of the preceding day.

- Second-morning urine samples will be collected and sent for analysis.

- Fasting blood samples will be drawn and sent for analysis.

- Weight and blood pressure will be measured.

- Compliance with the intake of the benfotiamine or placebo will be ensured by counting the pills in the last two visits of each patient.

## Withdrawal of individual subjects

Subjects can leave the study at any time for any reason if they wish to do so without any consequences. The investigator can decide to withdraw a subject from the study for urgent medical reasons.

### Specific criteria for withdrawal

- Serious adverse events
- Hospitalization of the patient
- Non compliance of the patient
- Change of concomitant medication which might influence the primary and secondary endpoints itself (e.g. ACE-inhibitors, AT II-antagonists)

## Replacement of individual subjects after withdrawal

Randomisation list, emergency envelopes and study medication will be prepared for 86 patients and another 86 patients for the case that the study goes on after interim analysis. If randomised patients are withdrawn from the study (drop-outs) then further patients will be admitted to the study until the scheduled sample size is achieved.

## Follow-up of subjects withdrawn from treatment

Subjects who withdraw from the study will be followed up according to the routine terms of care of diabetic patients attending in the clinic. Furthermore, any side effect or adverse reaction, which might or might not be a reason for withdrawal, will be reported and followed up carefully with the necessary actions.

## Premature termination of the study

The termination of the entire trial will be decided if a patient shows an unknown serious event that can be related to the study medication after thorough discussion. The decisions (individual or for the entire study) are taken by the sponsor and the investigators together.

# SAFETY REPORTING

## Section 10 WMO event

In accordance to section 10, subsection 1, of the WMO, the investigator will inform the subjects and the reviewing accredited METC if anything occurs, on the basis of which it appears that the disadvantages of participation may be significantly greater than was foreseen in the research proposal. The study will be suspended pending further review by the accredited METC, except insofar as suspension would jeopardize the subjects’ health. The investigator will take care that all subjects are kept informed.

## Adverse and serious adverse events

Adverse events are defined as any undesirable experience occurring to a subject during a clinical trial, whether or not considered related to the investigational drug. All adverse events reported spontaneously by the subject or observed by the investiga­tor or his staff will be recorded.
A serious adverse event is any untoward medical occurrence or effect that at any dose results in death;

- is life threatening (at the time of the event);
- requires hospitalization or prolongation of existing inpatients’ hospitalization;
- results in persistent or significant disability or incapacity;
- is a congenital anomaly or birth defect;
- is a new event of the trial likely to affect the safety of the subjects, such as an unexpected outcome of an adverse reaction, lack of efficacy of an IMP used for the treatment of a life threatening disease, major safety finding from a newly completed animal study, etc.

All SAEs will be reported to the accredited METC that approved the protocol, according to the requirements of that METC.

### Suspected unexpected serious adverse reactions (SUSAR)

Adverse reactions are all untoward and unintended responses to an investigational product related to any dose administered.
Unexpected adverse reactions are adverse reactions, of which the nature, or severity, is not consistent with the applicable product information (e.g. Investigator’s Brochure for an unapproved IMP or Summary of Product Characteristics (SPC) for an authorized medicinal product).
The sponsor will report expedited the following SUSARs to the METC:

SUSARs that have arisen in the clinical trial that was assessed by the METC;

SUSARs that have arisen in other clinical trial of the same sponsor and with the same medicinal product, and that could have consequences for the safety of the subjects involved in the clinical trial that was assessed by the METC.

The remaining SUSARs are recorded in an overview list (line-listing) that will be submitted once every half year to the METC. This line-listing provides an overview of all SUSARs from the study medicine, accompanied by a brief report highlighting the main points of concern.

The sponsor will report expedited all SUSARs to the competent authority, the Medicine Evaluation Board and the competent authorities in other Member States and to Wörwag Pharma GmbH & Co. KG.

The expedited reporting will occur not later than 15 days after the sponsor has first knowledge of the adverse reactions. For fatal or life threatening cases the term will be maximal 7 days for a preliminary report with another 8 days for completion of the report.

For all events that may require the unblinding of participant treatment, the medical monitor must be contacted before the unblinding occurs. In all cases, the medical monitor will notify the protocol chairs and the medical officer.
Any treatment unblinding will require a full written account. The account will include the reason for unblinding the study medication, the name of the medical monitor notified of the unblinding, and the date and time of the unblinding. The reasons for unblinding each individual study treatment should be included on the Treatment Unblinding Case Report Form and in the final study report. If a participant is unblinded, study therapy is discontinued and the participant is terminated from the study.

### Annual safety report

In addition to the expedited reporting of SUSARs, the sponsor will submit, once a year throughout the clinical trial, a safety report to the accredited METC, competent authority, Medicine Evaluation Board and competent authorities of the concerned Member States.

This safety report consists of:

- a list of all suspected (unexpected or expected) serious adverse reactions, along with an aggregated summary table of all reported serious adverse reactions, ordered by organ system, per study;

- a report concerning the safety of the subjects, consisting of a complete safety analysis and an evaluation of the balance between the efficacy and the harmfulness of the medicine under investigation.

## Follow-up of adverse events

All adverse events will be followed until they have abated, or until a stable situation has been reached. Depending on the event, follow up may require additional tests or medical procedures as indicated, and/or referral to the general physician or a medical specialist.

# STATISTICAL ANALYSIS

## Descriptive statistics

First, study patients will be described with their demographical and medical data. This will be done for the entire study population to describe study patients. Additionally this will be done separately for each of the two groups to investigate comparability of the groups (which might be disturbed due to drop-outs). Further, all outcome variables will be evaluated with descriptive statistical methods. For quantitative variables this includes maximum, median, mean, minimum as well as standard deviation. For qualitative variables absolute and relative frequencies will be used.

## Confirmatory testing

Primary outcome variables are the differences of β2-microglobulin and albumin in 24 hour urine between end and start of study medication. Both primary outcome variables will be tested with the t-test for independent samples. In case the distribution of the variables is clearly not a normal distribution, then the t-test will be replaced by the Wilcoxon test. To solve the problem of multiple confirmatory testing the p-values of the two tests will be adjusted by the Bonferroni-Holm procedure. The level of significance is established as 2.5% for the one-sided hypotheses excretion with verum is less at the end of the medication phase than excretion with placebo.

## Explorative analyses

Secondary and other outcome variables are tested as well and as far as it is promising. However, these tests are only explorative, not confirmatory, i.e. they generate new hypotheses. Further, subgroup analyses will be done with descriptive measures and explorative testing as far as interesting results are expected.

# ETHICAL CONSIDERATIONS

## Regulation statement

This study will be conducted according to the principles of the Declaration of Helsinki,adopted by the 18th WMA General Assembly, Helsinki, Finland, June 1964, and amended by the:
29th WMA General Assembly, Tokyo, Japan, October 1975,
35th WMA General Assembly, Venice, Italy, October 1983,
41st WMA General Assembly, Hong Kong, September 1989,
48th WMA General Assembly, Somerset West, Republic of South Africa, October 1996,
and the 52nd WMA General Assembly, Edinburgh, Scotland, October 2000.
Note of Clarification on Paragraph 29 added by the WMA General Assembly, Washington 2002
Note of Clarification on Paragraph 30 added by the WMA General Assembly, Tokyo 2004 ), and in accordance with the Medical Research Involving Human Subjects Act (WMO).

## Recruitment and consent

Subjects will be invited to participate in the study by sending letters to them, in which they will find a full explanation of the study, advantages and disadvantages of participating, and contact information of the research team members working on this study. Moreover, the letter contains contact information of independent physician, to whom subjects can address questions about the research before, during and after a study. The letter will be sent by the supervising doctor Prof. Dr. H.J.G. Bilo, internist nephrologist, and the patients will be given 2 weeks to consider their decision. The subjects are asked then to sign their written informed consent before they take part in the study. Furthermore, the patients will be requested to sign separate informed consents concerning their permission on storing small quantities of collected 24-hour urine (10 ml) and withdrawn blood (3 ml), in order to perform possible further laboratorial analyses which are not available at the current time or may turn out to be interesting (regarding diabetic nephropathy) in the frame of this research in the future. These materials will be stored in -80 ºC freezers at Isala Klinieken hospital in Zwolle. The permission of the medical ethical committee (METC) Zwolle will be requested before performing any of these possible laboratorial investigations. The general practitioners (Huisartsen) will be informed about the participation of their patients.

## Benefits and risks assessment, group relatedness

Benfotiamine, which is relatively free from adverse reactions, is going to be studied in order to prove its effect in prevention of the development of end-stage renal disease in susceptible diabetic patients. In case of positive effects on symptoms of diabetic nephropathy (albuminuria) a program on treatment of diabetic nephropathy by benfotiamine will be taken into account. Participation of diabetic nephropathy patients in this study is of great importance, and answering the questions of this clinical trial is not possible without observing and analyzing the parameters at the end of the study.

## Compensation for injury

The sponsor has a liability insurance which is in accordance with article 7, subsection 6 of the WMO.

The sponsor (also) has an insurance which is in accordance with the legal requirements in the Netherlands (Article 7 WMO and the Measure regarding Compulsory Insurance for Clinical Research in Humans of 23rd June 2003). This insurance provides cover for damage to research subjects through injury or death caused by the study.

1. € 450.000,-- (i.e. four hundred and fifty thousand Euro) for death or injury for each subject who participates in the Research;
2. € 3.500.000,-- (i.e. three million five hundred thousand Euro) for death or injury for all subjects who participate in the Research;
3. € 5.000.000,-- (i.e. five million Euro) for the total damage incurred by the organization for all damage disclosed by scientific research for the Sponsor as ‘verrichter’ in the meaning of said Act in each year of insurance coverage.

The insurance applies to the damage that becomes apparent during the study or within 4 years after the end of the study.

Subjects will be given written information about the taken out insurance when they participate in the study.

## Incentives

Participation of patients in the study is a free-will decision. Patients will not receive any financial support or priority for treatment of other diseases in the clinic during this clinical trial.

All costs that subjects need for transportation in order to attend in the clinic for the study purpose will be reimbursed completely.

# ADMINISTRATIVE ASPECTS AND PUBLICATION

## Handling and storage of data and documents

All data gathered from the subjects will be handled confidentially and anonymously.

A subject identification code list will be made to link the data to the subject in order to be able to trace data to an individual subject. This code will not be based on the patient initials and birth-date. The key to the code will be safeguarded by the investigator since the data will be kept for a longer period of time15 years. The handling of personal data will comply with the Dutch Personal Data Protection Act (in Dutch: De Wet Bescherming Persoonsgegevens, Wbp).

## Amendments

A ‘substantial amendment’ is defined as an amendment to the terms of the METC application, or to the protocol or any other supporting documentation, that is likely to affect to a significant degree:

- the safety or physical or mental integrity of the subjects of the trial;
- the scientific value of the trial;
- the conduct or management of the trial; or
- the quality or safety of any intervention used in the trial.

All substantial amendments will be notified to the METC and to the competent authority.

Non-substantial amendments will not be notified to the accredited METC and the competent authority, but will be recorded and filed by the sponsor.

## Quality control and Quality Assurance

The protocol requires that the investigators will permit trial-related monitoring, audits and reviews, and regulatory inspection(s), providing direct access to source data/documents.

Quality control will be ensured by regular visits of an independent person designated by the sponsor. All people participating in the investigation will participate before initiation of the trail to at least one common reunion where requirements for quality will be trained. The study will be conducted according to actual guidelines for GCP. All participating investigators are well trained and experienced in the investigation to be performed, the principal investigator has more than 15 years experience in conducting clinical trials.

## Annual progress report

The sponsor/investigator will submit a summary of the progress of the trial to the accredited METC once a year. Information will be provided on the date of inclusion of the first subject, numbers of subjects included and numbers of subjects that have completed the trial, serious adverse events/ serious adverse reactions, other problems, and amendments.

## End of study report

The sponsor will notify the accredited METC and the competent authority of the end of the study within a period of 90 days. The end of the study is defined as the last patient’s last visit.
In case the study is ended prematurely, the sponsor will notify the accredited METC and the competent authority within 15 days, including the reasons for the premature termination.
Within one year after the end of the study, the investigator/sponsor will submit a final study report with the results of the study, including any publications/abstracts of the study, to the accredited METC and the Competent Authority.

## Public disclosure and publication policy

This study is part of the EU-financed project PREDICTIONS with different participating parties. Before public disclosure, all knowledge generated in the project (e.g. pre-submission manuscript) will be submitted to project coordinator who will be requested to provide any recommendations for new patent application within 30 days. Where knowledge is to be protected, patent applications will be prepared by the concerned parties, according to their established practices.

In order to protect the confidential information, prior to submission for publication or presentation, the Publication Committee of PREDICTIONS shall provide each party 45 days to review any manuscript and 30 days to review any poster presentation, abstract or any other written or oral material which describes or discloses the results of the study. In addition, if one party requests in writing and demonstrates evidently that it is necessary to protect the rights of that party, the Publication Committee can withhold any publication or presentation an additional 45 days. Each party can demand to remove confidential information from a publication which could be damaging to party’s rights. The Publication Committee shall, in as far as it does not thereby compromise its scientific integrity, attempt to adapt its publication in such a way that party’s objections are met. Wörwag Pharma as manufacturer and the study medication will be named in any publication.

In case of results relevant for prophylactic or therapeutic intervention with benfotiamine in patients with diabetic nephropathy, Wörwag Pharma GmbH & Co. KG reserves the right of patent application nationally and internationally on his own behalf. Wörwag Pharma GmbH & Co. KG commits to announce the investigator(s) as inventors. The investigator(s) commit to render all statements and giving all signatures necessary for patent application towards authorities and patent offices. Wörwag Pharma GmbH & Co. KG bears all expenses in connection with patent application and grant of a patent. All rights and claims of the investigator(s) in connection with potential patents on the prophylactic of therapeutic use of benfotiamine in diabetic nephropathy are covered by the funding based on the PREDICTIONS contract. Other compensations by Wörwag Pharma are excluded.

Reference List

1. Zimmet P, Alberti KG, Shaw J. Global and societal implications of the diabetes epidemic. Nature 2001;414: 782-787.

2. Ritz E, Rychlik I, Locatelli F, Halimi S. End-stage renal failure in type 2 diabetes: A medical catastrophe of worldwide dimensions. Am J Kidney Dis 1999;34: 795-808.

3. Joyce AT, Iacoviello JM, Nag S et al. End-stage renal disease-associated managed care costs among patients with and without diabetes. Diabetes Care 2004;27: 2829-2835.

4. Fioretto P, Bruseghin M, Berto I, Gallina P, Manzato E, Mussap M. Renal protection in diabetes: role of glycemic control. J Am Soc Nephrol 2006;17: S86-S89.

5. D'Amico G. Tubulointerstitium as predictor of progression of glomerular diseases. Nephron 1999;83: 289-295.

6. Nath KA. Tubulointerstitial changes as a major determinant in the progression of renal damage. Am J Kidney Dis 1992;20: 1-17.

7. Fioretto P, Sutherland DE, Najafian B, Mauer M. Remodeling of renal interstitial and tubular lesions in pancreas transplant recipients. Kidney Int 2006;69: 907-912.

8. Effect of intensive therapy on the development and progression of diabetic nephropathy in the Diabetes Control and Complications Trial. The Diabetes Control and Complications (DCCT) Research Group. Kidney Int 1995;47: 1703-1720.

9. The effect of intensive treatment of diabetes on the development and progression of long-term complications in insulin-dependent diabetes mellitus. The Diabetes Control and Complications Trial Research Group. N Engl J Med 1993;329: 977-986.

10. Intensive blood-glucose control with sulphonylureas or insulin compared with conventional treatment and risk of complications in patients with type 2 diabetes (UKPDS 33). UK Prospective Diabetes Study (UKPDS) Group. Lancet 1998;352: 837-853.

11. Kawazu S, Tomono S, Shimizu M et al. The relationship between early diabetic nephropathy and control of plasma glucose in non-insulin-dependent diabetes mellitus. The effect of glycemic control on the development and progression of diabetic nephropathy in an 8-year follow-up study. J Diabetes Complications 1994;8: 13-17.

12. Steffes MW. Glomerular lesions of diabetes mellitus: preventable and reversible. Nephrol Dial Transplant 1999;14: 19-21.

13. Lewis EJ, Hunsicker LG, Bain RP, Rohde RD. The effect of angiotensin-converting-enzyme inhibition on diabetic nephropathy. The Collaborative Study Group. N Engl J Med 1993;329: 1456-1462.

14. Lewis EJ, Hunsicker LG, Clarke WR et al. Renoprotective effect of the angiotensin-receptor antagonist irbesartan in patients with nephropathy due to type 2 diabetes. N Engl J Med 2001;345: 851-860.

15. Brenner BM, Cooper ME, de Zeeuw D et al. Effects of losartan on renal and cardiovascular outcomes in patients with type 2 diabetes and nephropathy. N Engl J Med 2001;345: 861-869.

16. Waanders F, Greven WL, Baynes JW et al. Renal accumulation of pentosidine in non-diabetic proteinuria-induced renal damage in rats. Nephrol Dial Transplant 2005;20: 2060-2070.

17. Kuusniemi AM, Lapatto R, Holmberg C, Karikoski R, Rapola J, Jalanko H. Kidneys with heavy proteinuria show fibrosis, inflammation, and oxidative stress, but no tubular phenotypic change. Kidney Int 2005;68: 121-132.

18. Dalle-Donne I, Giustarini D, Colombo R, Rossi R, Milzani A. Protein carbonylation in human diseases. Trends Mol Med 2003;9: 169-176.

19. Bohlender JM, Franke S, Stein G, Wolf G. Advanced glycation end products and the kidney. Am J Physiol Renal Physiol 2005;289: F645-F659.

20. Chatziantoniou C, Dussaule JC. Insights into the mechanisms of renal fibrosis: is it possible to achieve regression? Am J Physiol Renal Physiol 2005;289: F227-F234.

21. Ishola DA, Jr., Post JA, van Timmeren MM et al. Albumin-bound fatty acids induce mitochondrial oxidant stress and impair antioxidant responses in proximal tubular cells. Kidney Int 2006;70: 724-731.

22. Hammes HP, Du X, Edelstein D et al. Benfotiamine blocks three major pathways of hyperglycemic damage and prevents experimental diabetic retinopathy. Nat Med 2003;9: 294-299.

23. Bakker SJ, Heine RJ, Gans RO. Thiamine may indirectly act as an antioxidant. Diabetologia 1997;40: 741-742.

24. Bakker SJ. Low thiamine intake and risk of cataract. Ophthalmology 2001;108: 1167.

25. Babaei-Jadidi R, Karachalias N, Ahmed N, Battah S, Thornalley PJ. Prevention of incipient diabetic nephropathy by high-dose thiamine and benfotiamine. Diabetes 2003;52: 2110-2120.

26. Thornalley PJ, Babaei-Jadidi R, Al AH et al. High prevalence of low plasma thiamine concentration in diabetes linked to a marker of vascular disease. Diabetologia 2007.

27. Eardley KS, Zehnder D, Quinkler M et al. The relationship between albuminuria, MCP-1/CCL2, and interstitial macrophages in chronic kidney disease. Kidney Int 2006;69: 1189-1197.

28. Grandaliano G, Gesualdo L, Ranieri E et al. Monocyte chemotactic peptide-1 expression in acute and chronic human nephritides: a pathogenetic role in interstitial monocytes recruitment. J Am Soc Nephrol 1996;7: 906-913.

29. Morii T, Fujita H, Narita T et al. Increased urinary excretion of monocyte chemoattractant protein-1 in proteinuric renal diseases. Ren Fail 2003;25: 439-444.

30. Prodjosudjadi W, Gerritsma JS, van Es LA, Daha MR, Bruijn JA. Monocyte chemoattractant protein-1 in normal and diseased human kidneys: an immunohistochemical analysis. Clin Nephrol 1995;44: 148-155.

31. Wada T, Furuichi K, Sakai N et al. Up-regulation of monocyte chemoattractant protein-1 in tubulointerstitial lesions of human diabetic nephropathy. Kidney Int 2000;58: 1492-1499.

32. Yoshimoto K, Wada T, Furuichi K, Sakai N, Iwata Y, Yokoyama H. CD68 and MCP-1/CCR2 expression of initial biopsies reflect the outcomes of membranous nephropathy. Nephron Clin Pract 2004;98: c25-c34.

33. Haupt E, Ledermann H, Kopcke W. Benfotiamine in the treatment of diabetic polyneuropathy--a three-week randomized, controlled pilot study (BEDIP study). Int J Clin Pharmacol Ther 2005;43: 71-77.

34. Stracke H, Lindemann A, Federlin K. A benfotiamine-vitamin B combination in treatment of diabetic polyneuropathy. Exp Clin Endocrinol Diabetes 1996;104: 311-316.

35. Schmidt J (2002) Wirksamkeit von Benfotiamin bei diabetischer Neuropathie. Breite Anwendungsbeobachtung unterstreicht Praxisbenefit. Kassenarzt 14/15, 40-43

36. Ledermann H., Wiedey KD (1989) Behandlung der manifesten diabetischen Polyneuropathie. Therapiewoche 39, 1445-1449

37. Stracke H et al. (1996) A benfotiamine-vitamin B combination in treatment of diabetic polyneuropathy. Exp Clin Endocrinol Diabetes 104: 311-316

38. Winkler G et al. (1999) Effectiveness of different benfotiamine dosage regimens in the treatment of painful diabetic neuropathy. Drug Res 49, 220-224

39. Hammes et al. (Data on file) Influence of benfotiamine on intracellular CML (carboxy-methyl-lysin)-accumulation in erythrocytes of type-II diabetic patients.

40. Lin J, Tan S, Liersch J et al (2001) Benfotiamin hemmt die intrazelluläre Bildung von Glykierungsfolgeprodukten in-vivo. Abstrct/Poster 107. Kongress DGEM Wiesbaden, 21.-25.04.2001

41. Stirban A, Negrean M, Horstmann T et al (2006) Benfotiamine prevents macro- and microvascular endothelial dysfunction and oxidative stress following a meal rich in advanced glycation end products in individuals with type 2 diabetes. Diabetes Care 29: 2064-2071

42. Stirban A, Negrean M, Stratmann B, Götting C, Salomon J, Kleesiek K, Tschoepe D (2007) Adiponectin Decreases Postprandially Following a Heat Processed Meal in People with Type 2 - an Effect Prevented by Benfotiamine and Cooking Method. Diabetes Care 30:0149-5992

43. Iber FL, Blass JP, Brin M, Leevy CM (1982) Thiamine in the elderly - relation to alcoholism and to neurological degenerative disease. Am J Clin Nutr 36:1067-1082

44. Expert group of vitamins and minerals (2000) Covering note for EVM/00/14/P - Review of Thiamin. August 2000

45. Food standards agency (2002) Draft report of the expert group on vitamins and minerals. August 2002
